# Feasibility of a Text Message–Based Alcohol Prevention Intervention for Parents of Rising Middle School Students: Randomized Controlled Trial

**DOI:** 10.2196/72823

**Published:** 2025-10-30

**Authors:** Marina Stranieri Pearsall, Melissa B Gilkey, Susan T Ennett, H Luz McNaughton Reyes, Nisha Gottfredson O'Shea

**Affiliations:** 1 Center for Behavioral Health and Well-being Research, RTI International, 3040 Cornwallis Rd, Durham, NC, 27709, United States, +1 9192481457; 2 Department of Health Behavior, Gillings School of Global Public Health, University of North Carolina at Chapel Hill, Chapel Hill, NC, United States

**Keywords:** adolescent, adolescent behavior, alcohol drinking, communication, mobile applications, prevention

## Abstract

**Background:**

Early-onset alcohol use (EOAU), or drinking before the age of 14 years, is a serious but highly preventable risk factor for later alcohol use. EOAU often begins at home, with sips of alcohol provided by parents. Few scalable interventions are available to engage parents in EOAU prevention.

**Objective:**

This study aimed to evaluate the feasibility of Better-Informed Parents Keeping Adolescents Safe From Alcohol (BIPAS Alcohol)*,* a digital family-based intervention for parents of rising middle schoolers.

**Methods:**

In 2023‐2024, we delivered BIPAS Alcohol to US parents (N*=*132) of 10- to 12-year-old children. The intervention consisted of a 3-month SMS text messaging curriculum and multimedia website. Guided by Bowen and colleagues’ framework, we surveyed parents to evaluate our intervention on its feasibility, including acceptability, integration, demand, and adaptation. We interviewed a subset of parents (n*=*11) to probe survey findings.

**Results:**

Parents rated BIPAS Alcohol highly on acceptability, with almost all agreeing the intervention kept their attention (117/123, 95.1%), offered useful information (121/123, 98.4%), and helped reduce chances of underage drinking (119/123, 96.7%). Most parents indicated plans to integrate the intervention into family life by referring to content in the future (113/123, 91.9%) or sharing content with others (107/123, 87.0%). In interviews, parents expressed high demand for SMS text messages, due to their short, “digestible” format, while finding the website more cumbersome. Although we designed the SMS text message curriculum for adults, some parents reported adapting the intervention by sharing texts with their children.

**Conclusions:**

Our digital family-based intervention demonstrated feasibility and warrants additional evaluation in a larger-scale trial with a wider audience.

## Introduction

Early-onset alcohol use (EOAU), or drinking before the age of 14 years, is a serious but highly preventable risk factor for later alcohol abuse that often begins at home, with sips of alcohol provided by parents [1-3]. By age 11, nearly one-quarter of US children have already sipped alcohol [4]. Children who engage in EOAU escalate from initiation to binge drinking and alcohol use disorder faster and more frequently than those who do not [5-10] and are at greater risk for drinking with peers, binge drinking, and alcohol-related harms [5-11]. Timing early-onset alcohol prevention before the transition to middle school is crucial because many students have already tried alcohol by the end of middle school and into high school.

Parents of rising middle schoolers are well-positioned to prevent EOAU. Parents are the primary source of alcohol-related information and socialization for children during the transition to middle school [12], are more influential than peers for children and young adolescents [13], and serve as children’s primary source of social modeling and information about alcohol use in the preteen years [14,15]. Research shows that many parents who provide alcohol have the mistaken belief that providing sips of alcohol teaches children how to drink in moderation; other parents report that they have not considered the risks [1].

Despite this evidence, existing universal prevention programs tend to be school-based or too late to prevent EOAU [16,17]. In addition, family-based interventions face the challenge of reaching parents and keeping them engaged. In-person family programs face dissemination and implementation barriers that limit reach at a broader level [18], such as difficulty obtaining transportation and childcare, busy schedules, and perceived stigma [19].

Programs are often not delivered in a mode that accommodates parents’ limited time. Existing programs, such as Strengthening Families Program and Talk, They Hear You, also incorporate parent-child communication skills and setting limits or rules with children [20,21], and Talk, They Hear You offers an array of resources online and in an app-based format for parents [21]. However, these programs are not designed to deliver small bites of information to accommodate busy schedules. An SMS text message–based intervention has the potential to address the need for a universal prevention strategy focused on families by overcoming many of the obstacles associated with traditional in-person prevention programs.

To address these gaps, we sought to develop and evaluate the feasibility of Better-Informed Parents Keeping Adolescents Safe From Alcohol (BIPAS Alcohol), a mobile, universal, family-based preventative pilot intervention designed to prevent early drinking onset that is delivered directly to parents of rising middle schoolers. The aims of this study were to assess acceptability, demand, integration, adaptation, and implementation according to the feasibility framework outlined by Bowen and colleagues [22]. By assessing the feasibility of this pilot intervention, this study lays the groundwork for larger-scale research to test the effectiveness of digital EOAU prevention interventions for families of adolescents.

## Methods

### Participants

Participants were parents of rising middle school children residing primarily in the Research Triangle area of North Carolina. Parents were eligible to participate if they: identified as a parent or guardian of a 10- to 12-year-old child with whom they lived at least 2 or more days a week; used any type of smartphone or other device with SMS text messaging capabilities; were able to complete study activities in English; and were willing to be randomized to receive the intervention immediately or after a 3-month waiting period. Parents who had more than one age-eligible child were asked to select the youngest child to be the primary focus of their study activities.

We recruited parents via electronic school newsletters delivered to parents of public school elementary students in Durham, Chatham, Charlotte-Mecklenburg, and Orange counties, participant referrals, social media posts, information disseminated through university listservs, social media posts, and local pediatric clinics. Among participants who reported their recruitment method, the majority were recruited through electronic school newsletters (69/132, 52.3%), participant referrals (29/132, 22.0%), and university listservs (22/132, 17.0%). Study advertisements instructed interested individuals to scan a QR code that directed them to an online prescreening questionnaire or to contact the study team via email. Research staff reviewed questionnaires and scheduled a phone call with parent-child dyads for further screening. During this phone call, research staff provided study details, confirmed eligibility, and described next steps for enrollment. Parents who wished to enroll completed an electronic consent form and a child permission form.

Recruitment occurred between May and November 2023. Enrolled participants were randomized with equal probability to receive the intervention immediately (n=69) or after a 3-month waiting period (n=63). Parents received the intervention between June 2023 and July 2024.

### BIPAS Alcohol Intervention

All parents who enrolled in our study received the BIPAS Alcohol intervention, which consisted of a 3-month SMS text message–based curriculum and a corresponding website. The study team developed content for BIPAS Alcohol from an existing evidence-based intervention entitled Mysteries, Max and Me (MMM) [23]. MMM is a family-based intervention that draws from social learning theory [24] to engage families in positive social modeling for youth alcohol prevention through print-based content delivered to parents of children in elementary school [23]. Children of parents who were randomized to receive MMM had less susceptibility to alcohol use at the 12-month follow-up (Cohen *d*=0.12). Mediation analyses showed that MMM reduced child susceptibility to alcohol use by increasing parental communication about family norms (Cohen *d*=0.16), peer influence (Cohen *d*=0.12), and media portrayals of alcohol (Cohen *d*=0.27), and by increasing alcohol-specific rule-setting (Cohen *d*=0.16) [1].

**Figure 1.**
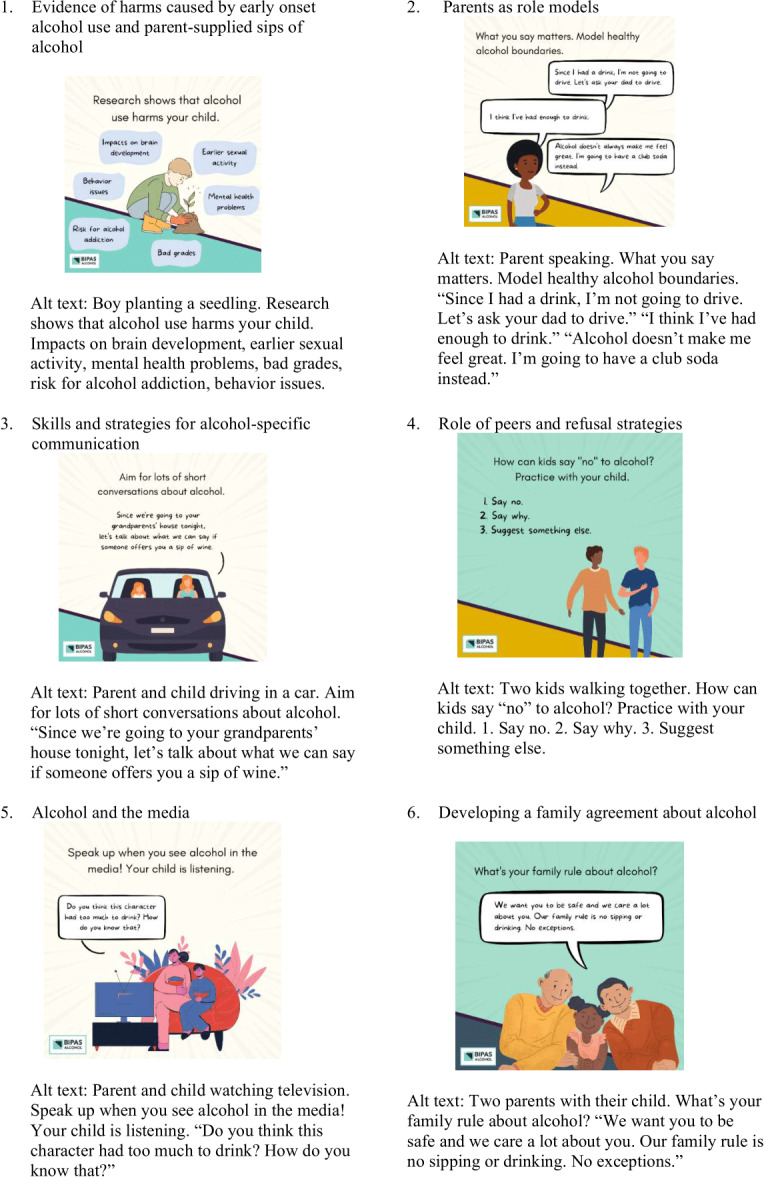
Examples of BIPAS Alcohol SMS text message graphics by theme.

**Figure 2.**
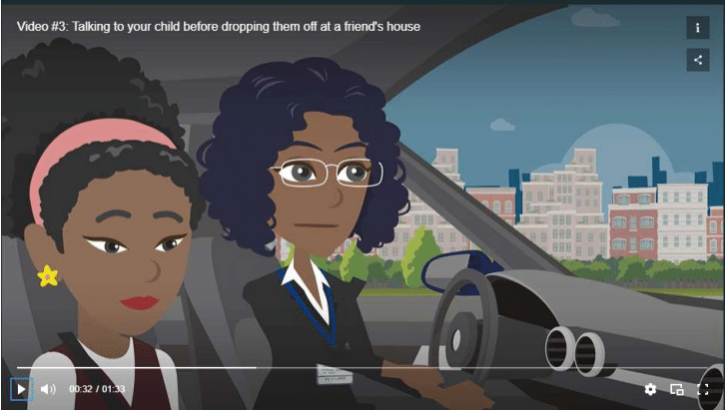
Example of BIPAS Alcohol video animation from the website.

MMM delivered study materials in printed booklets mailed to the homes of participating families. Our study team adapted MMM for use on a digital platform, including 50 brief SMS text messages containing comic-style graphics (Figure 1) and linked website content with video animations tailored to parents of rising middle school–aged children. Video animations depicted examples of conversations about alcohol in common situations, including a parent talking with their child about alcohol before dropping them off at a friend’s house (Figure 2), noticing someone visibly drunk at a family gathering, and watching a movie with depictions of young people drinking. Our team also targeted messages to 2 parent groups for universal delivery: parents identifying as drinkers and nondrinkers.

Content was designed to be engaging, informative, and visually appealing. BIPAS Alcohol’s core message encouraged parents not to allow their children to sip alcohol by fostering discussions and family rules about alcohol. Message content proceeded along the following progression of themes: (1) evidence of harms caused by EOAU and parent-supplied sips of alcohol; (2) parents as role models; (3) skills and strategies for alcohol-specific communication, including setting clear, alcohol-specific rules with children; (4) role of peers and refusal strategies; (5) alcohol in the media; and (6) developing a family agreement about alcohol. Consistent with these themes, messages focused on what the research says about alcohol and why kids drink (20 messages), sipping (8 messages), media (7 messages), friends and peers (7 messages), and family rules (8 messages). We designed messages to be accessible for parents of lower literacy, with more than half (31/50, 62%) of SMS text messages including a graphic. Messages had a reading ease score of 71.8 and grade level of 6.3 (Flesch-Kincaid), and visual depictions of families in animations and graphics were purposefully designed to be diverse with regard to race and ethnicity and family structure. Two groups of messages were tailored to parents who use and do not use alcohol to appeal to diverse audiences.

During the development process, we assessed SMS text messages iteratively with cognitive interviews with 11 parents of rising middle school children who did not subsequently participate in the intervention. These parents provided feedback on the content, appearance, tone, acceptability, and persuasiveness of our messages.

The intervention website included a homepage with an animated welcome video and individual pages corresponding to intervention themes (“Sipping Alcohol,” “Talking the Talk,” “Friends,” “Media,” and “Family Agreement”). The website also featured an “About” page with information about our study team, “FAQs” anticipating parent questions about parent alcohol use, sipping, alcohol use in cooking, and religious use, and “Resources” with resources for adults struggling with alcohol. Four animations on the website ranged from 79 seconds to 104 seconds in length. Parents were directed to specific pages on the website through SMS text message links throughout the intervention.

### Data Collection Procedures

#### Study Design

After enrollment, participants were randomized using a random number generator with equal probability to an immediate intervention (n=69) or a waitlist control group (n=63). Participants and study team members were not blinded to the study assignment groups, as it was clear which group participants were placed in once enrolled. This study will draw on feasibility data (measures described below) from both groups of participants during their active intervention (n=132). Outcomes between groups will be compared elsewhere.

#### Parent Surveys

Parents completed web-based surveys assessing parents’ attitudes, beliefs, and behaviors at baseline and 3 months (intervention completion). We texted parents with invitations to complete the surveys, followed by up to 2 SMS text messages or email reminders for nonrespondents. Parents received a US $25 gift card incentive after completion of each survey. Survey response rates were 100% at baseline (N=132) and 93.2% at 3 months (n=123).

#### Parent Interviews

Parents indicated in their 3-month survey whether they were interested in participating in qualitative interviews, and study staff reached out to the first 17 interested parents. Eleven parents completed interviews, which ranged from 32 to 55 minutes in length. A trained member of the study team conducted interviews using a semistructured interview guide designed to explore parents’ perspectives about the intervention and their experiences participating. We audio-recorded and transcribed interviews with permission. Parents received a US $40 gift card incentive for completing an interview.

### Measures

We developed our measures using Bowen and colleagues’ feasibility framework, which emphasizes the importance of intervention acceptability, demand, integration, adaptation, and implementation [22]. In the baseline and 3-month surveys, feasibility was assessed with survey items using a 4-point response scale ranging from “1” (strongly agree) to “4” (strongly disagree). The definition of our measures and their operationalization is detailed in Table 1.

**Table 1.** Operationalization of feasibility measures.

Feasibility measures	Definition	Operationalization
Acceptability	Participants’ satisfaction with BIPAS Alcohol*^a^*.	Six survey items assessed BIPAS Alcohol was useful, helpful, kept parents’ attention, had an appropriate length, will help reduce child’s chances of underage drinking, and the extent to which parents knew what BIPAS Alcohol was trying to do.^b^ Qualitative interviews assessed parents’ perspectives on content, SMS text messages, and the website.
Demand	Parents’ actual use of BIPAS Alcohol.	Website analytics detailed the number of visits per page and average time spent per page. Qualitative interviews assessed materials that parents did or did not use.
Integration	Extent to which BIPAS Alcohol integrated into the existing familial system.	Two survey items assessed whether parents planned to refer to the content in the future^b^ or plan to share it with others.^c^ Qualitative interviews assessed behavior changes, parents’ parenting or planned parenting changes, and how parents involved their children in the intervention.
Adaptation	Extent to which changes were made to BIPAS Alcohol by study participants.	Qualitative interviews assessed whether parents made modifications to the program or engaged with program materials in a way not anticipated by the study team. Interviews also assessed how parents shared program materials with other adults or children not directly targeted by the intervention.
Implementation	Extent to which BIPAS Alcohol can successfully be delivered to the intended audience.	Study retention rate and study team documentation of technical problems or recruitment issues over the course of delivering the intervention, including the enrollment rate and recruitment rate.

a BIPAS: Better-Informed Parents Keeping Adolescents Safe From Alcohol.

b Survey response scale ranged from “1” (strongly agree) to “4” (strongly disagree).

c Survey response scale ranged from “1” (very likely) to “4” (not at all likely).

### Data Analysis

We analyzed quantitative survey data using descriptive statistics and qualitative interview data via thematic content analysis [25]. Two authors (MSP and MBG) developed a codebook based on feasibility measures. After independently applying the codes to 2 transcripts, the authors met and discussed the codes for each response. Discrepancies were resolved through discussion, and the codebook was refined. Afterward, 1 author (MSP) coded all 11 transcripts and analyzed each code to determine themes.

### Ethical Considerations

The study protocol was approved by the University of North Carolina at Chapel Hill Institutional Review Board (study #22‐0101). The study protocol and results are available on ClinicalTrials.gov (ID #NCT05520333). Written informed consent was obtained electronically through Qualtrics from all parents before participation. Participant privacy and confidentiality were rigorously maintained throughout the study with strict adherence to a data safety and monitoring plan. All data were deidentified and stored on secure, password-protected servers with access restricted to institutional review board–approved study personnel. Participants received a US $25 electronic gift card after completion of each study survey and a US $40 gift card for completing an interview. No identifiable information is presented in this manuscript.

## Results

### Participant Characteristics

**Figure 3.**
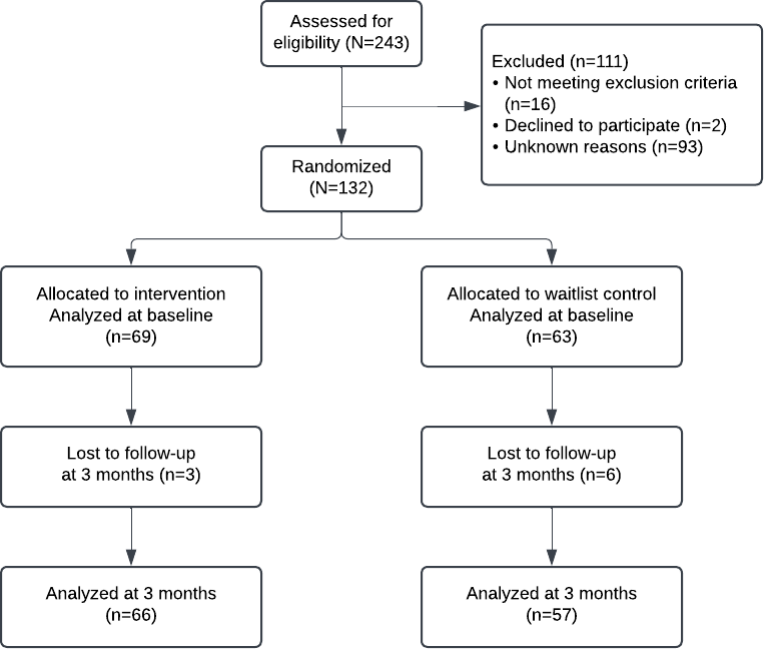
Flow of participants through BIPAS Alcohol intervention.

Most parents in our sample were mothers (115/132, 87.1%), identified as non-Hispanic White (111/132, 84.1%), and had high educational attainment (90/132, 68.2% reported a master’s degree or above, Table 2). The subset of parents who participated in qualitative interviews had similar characteristics. Our demographic sample reflects the geographic area as well as methods of recruitment (eg, university listserv). Of 243 parents screened, 227 parents were eligible, and 132 parents (58.1%) consented and enrolled (Figure 3). While we do not have direct feedback from parents who did not enroll, coordinating a time to talk to a study team member with their child present was likely the biggest hurdle to enrollment in the screening process.

**Table 2.** Parent participant characteristics.

Characteristics	Survey (n=132), n (%)	Interview (n=11), n (%)
Relationship to child		
Mother	115 (87.1)	10 (90.9)
Father	16 (12.1)	1 (9.1)
Guardian	1 (0.8)	0 (0)
Age (years)		
≤40	37 (28.0)	4 (36.4)
41‐45	48 (36.4)	2 (18.2)
46 and older	47 (35.6)	5 (45.5)
Race and ethnicity		
Non-Hispanic White	111 (84.1)	11 (100)
Asian	8 (6.1)	0 (0)
Black or African American	3 (2.3)	0 (0)
Hispanic or Latino	2 (1.5)	0 (0)
More than 1 race or another race	8 (6.1)	0 (0)
Highest degree earned		
Master’s, doctorate, or professional degree	90 (68.2)	7 (63.6)
Bachelor’s, associate’s, or technical degree	35 (26.5)	3 (27.3)
High school or less than high school	7 (5.3)	1 (9.1)
Parent drinks alcohol		
Yes	103 (78.0)	9 (81.8)
No	29 (22.0)	2 (18.2)
Alcohol intake over the past 30 days		
4+ times per week	16 (12.1)	1 (9.1)
2‐3 times per week	31 (23.5)	2 (18.2)
2‐4 times per month	29 (22.0)	4 (36.4)
Monthly or less	24 (18.2)	2 (18.2)
Never	32 (24.2)	2 (18.2)

### Acceptability

Survey results showed that BIPAS Alcohol was acceptable to parents (Table 3). Parents overwhelmingly agreed that the program kept their attention (117/123, 95.1% of parents strongly agreed or agreed at 3 months) and that they learned useful information (121/123, 98.4% of parents strongly agreed or agreed at 3 months). Notably, 96.7% (119/123) of parents reported that the program would help them reduce their child’s chances of underage drinking at 3 months. Parents found the SMS text messages to be the most helpful component of the program, followed by the website and videos.

**Table 3.** Parents’ perspectives about BIPAS Alcohol at 3 months (n=123).

Parents’ perspectives^a^	Strongly disagree or disagree, n (%)	Agree or strongly agree, n (%)
Survey kept attention	6 (4.9)	117 (95.1)
Program was too long	101 (82.1)	22 (17.9)
Learned useful information	2 (1.6)	121 (98.4)
Will help me reduce my child’s chances of underage drinking	4 (3.3)	119 (96.7)
Program covered topics that are important to me	0 (0)	123 (100)
Understand what program is trying to do	0 (0)	123 (100)
Time well spent	2 (1.6)	121 (98.4)
Will refer to content in future	10 (8.1)	113 (91.9)
Likely to share content with others	16 (13.0)	107 (87.0)

a Survey data of parents from both intervention and waitlist control groups during the active intervention period.

In qualitative interviews, parents discussed that the program was acceptable, useful, convincing, and liked the short, “digestible” format of the program (Table 4). Parents reported that they had not thought about talking with their children about alcohol prior to receiving the BIPAS Alcohol intervention, but reported that the intervention provided both new information and an awareness around the need to talk about alcohol with their child now. One parent remarked that BIPAS Alcohol “opened my eyes to how many influences there are” (eg, social media, movies, advertisements, parents and other caregivers, peers, and parents of peers).

**Table 4.** Selected quotes by theme from qualitative interviews.

Themes	Quotes
Parents had positive feelings about the intervention	It changed my behavior with my daughter in a way that was very easy. That will hopefully make a difference and it’s something that I don’t know. I know I can go back and read and find information as she gets older and [her] needs change.
The program brought up topics that parents had not previously considered talking to their child about	I think it gave me an excuse to think concretely about things that I wouldn’t have otherwise. [The program] has given us really a jumping point to talk more about issues I thought were further away in the future for us and I wouldn’t have brought it up on my own. So I’m appreciative of that because it’s just given us some talking points… …It just hadn’t occurred to me to talk to my daughter who’s in elementary school still about alcohol…It was a really good reminder [that], no, actually I can’t just assume that she is going to read my mind and know how I feel about these things.
Parents liked that the program was brief	The format was very short nuggets [of information]…It wasn’t hard to digest at all. The message was very short for short attention span people. I really liked [the text messages] because they were small amounts of snackable information. If they hit me at the right time, they could prompt interesting discussions. I liked that it held my attention just for chunks at a time. I didn’t have to spend a half hour having a conversation when we got the graphic. We could have a quick two or three minute conversation, and I think that really held everybody’s interest.
Parents found program content convincing or needed additional information	After the first part of the intervention, I didn’t need to be convinced. I was convinced. I was like, oh, okay, I’m going to make sure of that. [The program] really opened my eyes to how many influences are out there as regards to alcohol and adolescents. I’m a parent that strongly believes I would much rather my child drink at home where she’s safe…the study really opened my eyes just like, oh, hey, that’s kind of wrong. You shouldn’t do that. So one of the things I struggle with a bit in the messaging is this sort of hard and fast rule of you just don’t drink period.
Parents found the program content helpful in talking about alcohol	It brought up an opportunity to talk about [alcohol] that wasn’t accusatory or in reaction to a negative incident…it just gave a neutral platform to talk about it. I think the information both gave me the impetus to initiate the conversation and the security or the confidence that the conversation doesn’t need to be very elaborate. I liked [the program] and it sparked a lot of conversation at home, but also with fellow parents. I think it’s been helpful because they’re easy conversation starters and it doesn’t have to be a special conversation set aside during the day. It can just happen anytime. It felt very fluid and natural.
Parents liked the format of the program	I particularly [liked] the text messages…a short but sweet punch of information…time is short often, so anything that cuts to the chase of the most pertinent tidbits I think were what spoke to me the most. I am in favor of anything that gives me a structure to hang things on that I’ve always known I should do, but never quite known how to approach. And I think this program did that well.
Time constraints prevented parents from reviewing all the program content	I was less likely to read through a long [website] page than I was a more condensed text message, but it was still useful information.

The short, succinct format of BIPAS Alcohol messages stood out to parents (Table 4). Parents said the program “wasn’t hard to digest” and liked the “snackable” messages. Practical examples and talking points helped parents to initiate conversations with children, and parents appreciated ideas about how to approach these conversations. Parents described how helpful it was to think about BIPAS Alcohol’s approach of having multiple, shorter conversations with children as opposed to a “one and done” talk about alcohol. That the information came from a neutral, university-affiliated third party was also cited by parents as enabling trust.

As this program targeted rising middle school-aged children, parents brought up the age appropriateness of the content; some said that the program material was age-appropriate for their child, and others reported that they had initially thought their child was too young, but changed their mind after receiving more information from the BIPAS Alcohol messages. Several parents said that they had not realized how much their child had already been exposed to alcohol or, alternatively, appreciated that they were receiving this information proactively.

Parents expressed conflicting views about both the perceived repetitiveness in BIPAS Alcohol’s messaging and the program’s stance on avoiding all alcohol for children. This included some mixed feelings about the program’s core message around not allowing sipping, including uncertainty that sipping would lead to drinking for children. Although the information was available on the website, some parents requested additional evidence to counteract the cultural narrative that drinking in moderation was healthy, even for underage children. Parents who reported that they did not engage much with BIPAS Alcohol’s website and animations found this content too long or time-consuming to access. Most parents did not watch all the animations, and some did not find them relatable to their family or felt they did not have time to watch them. However, parents believed the website was an important component of the intervention, for the legitimacy and credibility of the program and to refer to for additional resources.

The SMS text message portion of the program was many parents’ favorite part due to the short, concise format and engaging graphics, and parents reported positive feelings about texts. Parents liked the graphics and highlighted that they helped capture their attention or communicate with their child.

### Demand

Website analytics during the active intervention showed that the homepage received the most page views (n=610 views), followed by the “Talking the Talk” page (n=467) and “Sipping Alcohol” page (n=242). The “Resources” page received the least number of views (n=124). Website visitors spent the most time on the “Talking the Talk” page (average 3.3 min), followed by the “Media” (average 2.7 min) and “Sipping” pages (average 2.2 min). Visitors spent the least amount of time on the “About” page (average 1 min). In qualitative interviews, parents reported reading all or most SMS text messages they received. Parents also reported visiting the website, although with greater variance: some visited only a few times during the intervention, while others visited weekly or more often. Parents noted that time constraints made it difficult to frequently follow links to the website or watch animations.

Parents who reported that they did not engage much with BIPAS Alcohol’s website and animations found this content too long or time-consuming to access. Most parents did not watch all the animations, and some did not find them relatable to their family or felt they did not have time to watch them. Parents believed the website was an important component of the intervention; however, for the legitimacy and credibility of the program and to refer to for additional resources.

### Integration

In the 3-month surveys, 91.9% (113/123) of parents agreed or strongly agreed that they would refer to this content in the future, while 87.0% (107/123) of parents said that they are somewhat likely or very likely to share this content with others.

In qualitative interviews, all parents reported that they had talked to their child about alcohol and discussed family rules about alcohol after participating in BIPAS Alcohol. Two themes that emerged were that the program helped open new conversations with children and enabled multiple, smaller conversations, including prompts to have these types of conversations. The program, according to 1 parent, “gave me the impetus to initiate the conversation and the security or the confidence that the conversation doesn’t need to be very elaborate.”

Parents appreciated suggested scenarios focused on talking with their children about alcohol and cited that the program helped them have new conversations with their children within existing family life—such as around a family history with alcohol, observations of alcohol in media or movies, and navigating alcohol in gatherings and holidays with family and friends. Two parents described the program as a starting point to broader conversations with their children around other substances. Said 1 parent: “It has given us a jumping point to talk more about issues I thought were further away in the future for us and I wouldn’t have brought up on my own.”

Parents also discussed making personal behavior changes or parenting practices around alcohol, such as no longer asking their child to bring them drinks, modifying the presence or accessibility of alcohol in their home, or no longer allowing children to have sips of alcohol or mocktails. Several parents described their own mindset changes around alcohol, such as decreasing personal consumption altogether or in front of their child. Parents reported initiating conversations with other adults about alcohol, although some did not, with 1 parent noting that this topic was “touchy.” Two parents wondered about the developmental time span through which BIPAS Alcohol’s message regarding the importance of children abstaining from alcohol completely would continue to be relevant to their child.

### Adaptation

In qualitative interviews, parents discussed ways they adapted the program. Parents shared that they shared program materials directly with their child as part of alcohol-specific conversations, which the study team had not anticipated. Some parents also described incorporating other children not targeted by BIPAS Alcohol in the intervention, either in alcohol-specific discussions or by sharing program materials with their child’s sibling or peers. Other parents noted broadening conversations about alcohol to other substances, such as illegal and legal drug use, smoking, vaping, and how to handle alcohol as their child gets older.

### Implementation

A retention rate of 93.2% (123/132) was calculated as the percentage of participants who completed the 3-month survey. The vast majority of SMS text messages were delivered as planned. However, 1 technical issue occurred on the SMS text delivery platform, where SMS text messages were temporarily capped, blocking SMS text message delivery to 31 participants. This issue was discovered and fixed, and parents continued to receive SMS text messages. Parents did not report any technical issues during the intervention.

### Suggested Changes to Strengthen the Program

In interviews, parents were invested in the success of future iterations of the program and shared many ideas for strengthening it. Some parents wanted more guidance about how to modify discussions and rules with kids as they got older and were exposed to more alcohol. For example, parents mentioned wanting content geared toward drinking and driving, texting and driving, and preparing for children to leave home or enter college. Noted one parent: “I feel like there does need to be a conversation about how to transition my kid into eventually trying alcohol.” Another parent questioned, “I think that the guidance and the rules that we’ve talked about for a younger age group just aren’t the right rules for a 16-year-old. And so how do you get there?”

Parents shared ideas for broadening the program’s content focus. Parents sought guidance around other substances, such as legal and illegal drugs and tobacco, or topics such as peer pressure and public health context. Similarly, some parents wanted additional information on cultural differences around alcohol, either within different households or across different countries.

Parents suggested additional content that would be helpful, including additional scenarios for graphics, discussion about other alcohol-related themes, such as secrecy or details about where kids may have access to alcohol, and additional information on the science of brain development. Parents were also interested in different options for guidance based on parenting style and more interactive activities for parents and kids. Several parents wanted additional references to justify the program’s recommendations or resources for parents or other family members struggling with alcohol dependency, which were both available on the website but were not accessed by all parents.

## Discussion

### Principal Findings

Results from the BIPAS Alcohol pilot indicate that our SMS text message–based intervention is a feasible way to engage parents in EOAU prevention for rising middle schoolers. Measures of acceptability were high, and parents shared that they found the intervention appealing, engaging, and helpful. Although some parents were initially concerned about whether the intervention was age-appropriate for their 10‐ to 12-year-old child, they concluded that the content was appropriate after participating in the intervention. This is a noteworthy finding given evidence of some parents’ reluctance to initiate early conversations about alcohol use with their children [3].

Parents also reported high demand and integration. In particular, parents’ usage of SMS text messages was very high, which corresponds to prior studies showing the usefulness of SMS text messages for intervention delivery [26,27]. SMS text messages offer a promising method of overcoming the limitations of existing interventions, including the original MMM study, to make a more highly scalable intervention. Furthermore, parents reported integration by applying content primarily by initiating conversations about alcohol with their children. This suggests promise for effectiveness, which we will examine in a separate study.

### Strengths and Limitations

Our pilot lays the foundation for future research, but findings should be interpreted in light of limitations. The sample was comprised of relatively affluent, white parents within a relatively small geographic area, which limits generalizability. Although our qualitative interviews included a diversity of parents identifying as drinkers and nondrinkers, our sample was not racially or ethnically diverse. Of note, however, white and highly educated adults also have the highest rates of alcohol consumption and permissive sipping behaviors with underaged children [2,28], so this approach resulted in targeting the highest risk groups. Furthermore, over-representation of mothers in parenting interventions is common [29,30] and may reflect gendered parenting differences with respect to which parents spend more time managing communication with children and other caregivers. In addition, we relied on parental reports to assess the demand for SMS text messages and were not able to otherwise determine whether participants read SMS text messages from available data; these and other novel measures will require validation in a future study. Although parents perceived BIPAS Alcohol as effective, we plan to evaluate its effectiveness in a future study. Finally, this study’s follow-up was limited to 3 months; longer-term outcomes and data from children also need to be considered to understand effectiveness in preventing EOAU.

### Future Directions

Next steps for BIPAS Alcohol include evaluating this intervention in a larger trial to ensure that it is feasible and efficacious in preventing children from drinking in a larger, more diverse sample. We also plan to expand BIPAS Alcohol’s reach to ensure it is available to parents who prefer Spanish and to incorporate additional content in response to the qualitative feedback noted in this study. Finally, a larger trial will provide the opportunity to explore how the impact of BIPAS varies across subpopulations of parents and according to seasonal changes in alcohol availability due to holidays, summer vacations, and the like.

### Conclusions

The BIPAS Alcohol SMS text message–based parenting intervention demonstrated feasibility in a sample of 132 parents. Parents participating in this intervention reported that they found the intervention acceptable, useful, and easy to integrate into their family. Future research is needed to revise the SMS text message content for a larger scale trial and test this intervention for generalizability to a wider audience.

## Supplementary material

10.2196/72823 Checklist 1 CONSORT checklist.

## Abbreviations

Alcohol: Better-Informed Parents Keeping Adolescents Safe From Alcohol
EOAU: early-onset alcohol use
MMM: Mysteries, Max and Me
